# S100P Expression Is a Novel Prognostic Factor in Hepatocellular Carcinoma and Predicts Survival in Patients with High Tumor Stage or Early Recurrent Tumors

**DOI:** 10.1371/journal.pone.0065501

**Published:** 2013-06-13

**Authors:** Ray-Hwang Yuan, Ko-Tung Chang, Yu-Ling Chen, Hey-Chi Hsu, Po-Huang Lee, Po-Lin Lai, Yung-Ming Jeng

**Affiliations:** 1 Department of Surgery, National Taiwan University Hospital and College of Medicine, National Taiwan University, Taipei, Taiwan; 2 Department of Surgery, National Taiwan University Hospital, Yun-Lin Branch, Yun-Lin, Taiwan; 3 Department of Life Science, National Pingtung University of Science and Technology, Pingtung, Taiwan; 4 Graduate Institute of Pathology, National Taiwan University, Taipei, Taiwan; 5 Department of Pathology, National Taiwan University Hospital, Taipei, Taiwan; Peking Union Medical College Hospital, Peking Union Medical College, Chinese Academy of Medical Sciences, China

## Abstract

The calcium-binding protein S100P is expressed in a variety of human cancer cells and is important in cancer cell growth and invasion. Using differential display, we found S100P is overexpressed in human hepatocellular carcinoma (HCC). We examined the expression of 305 unifocal, primary HCC tumors using immunohistochemistry. The S100P protein was expressed in 173 of the 305 (56.7%) HCC tumors. The expression of S100P correlated with female sex (*P = *0.0162), high serum α-fetoprotein level (*P = *0.0001), high tumor grade (*P = *0.0029), high tumor stage (*P = *0.0319), the presence of the *p53* mutation (*P = *0.0032), and the absence of the *β-catenin* mutation (*P = *0.0489). Patients with HCC tumors that expressed S100P were more likely to have early tumor recurrence (ETR) (*P = *0.0189) and lower 5-year survival (*P = *0.0023). The multivariate analysis confirmed that S100P expression was an independent prognostic factor in HCC. The combinatorial analysis showed an additive unfavorable prognostic interaction between S100P expression and the *p53* mutation. In contrast, the *β-catenin* mutation was associated with better prognosis in both S100P-positive and -negative HCCs. Furthermore, S100P expression was a predictor of survival in HCC patients with high tumor stage or ETR (*P = *0.0026 and *P = *0.0002, respectively). Our study indicates the expression of the S100P protein is a novel independent predictor for poor prognosis in HCC, and it is also an unfavorable prognostic predictor in HCC patients with high tumor stage or ETR.

## Introduction

Hepatocellular carcinoma (HCC) is one of the most common cancers worldwide, particularly in Taiwan, southern China, Southeast Asia, and sub-Saharan Africa, and the incidence of HCC is increasing in Western countries [1]. The major risk factors for HCC are hepatitis B and C, cirrhosis, and exposure to environmental carcinogens, such as aflatoxin [2]. Although surgical resection and various methods of tumor ablation can be curative or prolong survival, the outcome for patients with HCC remains grave. This is particularly true in advanced-stage HCC because the tumor has often spread throughout the liver via the intrahepatic portal venous system, and a considerable number of HCC patients develop early intrahepatic and/or extrahepatic recurrence postoperatively [3].

Early tumor recurrences (ETRs) arise primarily from intrahepatic metastases, and a significantly poorer prognosis [4]. However, the molecular factors related to tumor progression and ETR in HCC remain unclear. Therefore, the identification of molecular markers that correlate with tumor progression, ETR, and poor prognosis would aid efforts to establish better treatment plans for HCC patients.

One way to elucidate the pathogenesis of cancers is to identify the genes that are up- and down-regulated during the disease course. Methods for detecting such altered expression include mRNA differential display (DD), serial analysis of gene expression, subtractive hybridization, proteomics, and cDNA microarray. Using DD, we identified several upregulated genes in HCC that have clinical significance with regard to tumor proliferation and metastasis [5]–[11]. These include KIAA0101, stathmin, Reg1A/PAP, and CK19. We also identified a cDNA clone that is identical to *S100P*, which is preferentially expressed in tumors.

The S100 proteins are small, dimeric members of the EF-hand superfamily of Ca^2+^-binding proteins that contain 2 Helix-E and Helix-F loop-hand Ca^2+^-binding motifs that mediate Ca^2+^-dependent signal transduction. They play important roles in many intracellular and extracellular processes, including the regulation of protein phosphorylation, enzyme activation, gene transcription, the assembly of cytoskeleton components, cell proliferation and differentiation [12]. The S100P protein has been reported to be located in the nucleus, in the cytoplasm, at the cell membrane, and in the extracellular space, depending on the experiment conditions [13]–[15]. The S100P protein functions as both an extracellular and intracellular signaling molecule. In the extracellular space, S100P interacts with the receptor for advanced-glycation end products to activate signal transduction pathways, including the mitogen-activated protein kinase, serine protein kinase, extracellular-regulated kinase, and nuclear factor pathways [16]–[18], and promote tumor development [19]. Intracellular S100P interacts with the cytoskeletal multidomain protein ezrin through a Ca^2+^-dependent mechanism [20]. Ezrin links the plasma membrane and the actin cytoskeleton to regulate cell migration and cell proliferation [21]. Another binding partner of S100P is CacyBP/SIP. The interaction of S100P and CacyBP/SIP leads to the degradation of β-catenin [22]. Thus, S100P contributes to cancer progression by promoting cell proliferation, cell survival, angiogenesis, and metastasis.

The S100P protein was first identified in the human placenta [23], and has been reported to be overexpressed in multiple types of cancer cells, including breast cancer [13], [24], esophageal cancer [15], colon cancer [19], lung cancer [25], pancreatic cancer [26], [27], prostatic cancer [28], ovarian cancer [29], cholangiocarcinoma [30]–[32], and HCC [33]. However, the clinical and pathological significance of S100P expression in human HCC remains unclear. Moreover, HCC harbors frequent genetic mutations in *p53* and *β-catenin*, which have opposing roles in tumor progression, ETR, and prognosis [4], [34], [35]. The mutations of *p53* are associated with more advanced HCC and poor prognosis [4], [35], whereas the mutations of *β-catenin* are associated with less invasive tumors and better prognosis [34]. The expression of S100P in conjunction with these critical gene mutations requires further investigation to better understand the role of S100P in HCC progression. The aims of our study were to elucidate the role of S100P in vascular invasion, ETR, and HCC prognosis, to investigate the relationships between S100P expression and the *p53* and *β-catenin* mutations, and to evaluate S100P as a predictive biomarker for survival in HCC patients with high tumor stage or ETR.

## Materials and Methods

### Tissue Samples

A total of 305 unifocal, primary HCC tumors surgically resected from patients at National Taiwan University Hospital from 1982 to 1998 were used in this study. All resected tumors underwent detailed pathological assessment, and all patients received regular follow-up examinations, as described previously [5], [6]. Our study was approved by the Ethics Committee of National Taiwan University Hospital (approval no. 201107042RC), and all study procedures were conducted therein. All study participants provided written informed consent, which was approved by the Ethics Committee of National Taiwan University Hospital. One teenaged male was included in our study, for whom written, informed consent was obtained from his parents. There were no other minor participants in our study. The anonymity of all patients was maintained, and all specimens were analyzed in a blinded manner. The HCC patients included 239 males and 66 females, with a mean age of 55.09 years (range, 15 to 88 years). Serum hepatitis B surface antigen (HBsAg) was detected in 202 of 305 (66.2%) cases, and hepatitis C antibody (anti-HCV) was detected in 97 of 281 (34.5%) cases, 20 of which were positive for both. All patients had adequate liver function reserve at the time that they received curative liver resection, and their records contained complete clinical, histopathological, and follow-up data. No patients had distant metastasis, nor had they received anticancer treatments before undergoing surgery, such as transhepatic arterial embolization, percutaneous ethanol injection, radiofrequency ablation, or chemotherapy.

### Histology and Tumor Staging

Surgically resected specimens were formalin fixed and paraffin embedded. Histological sections were cut at 5-µm thickness and stained with hematoxylin-eosin. All specimens were reviewed by the same investigator (HCH) to determine tumor grade and stage. The tumor grading was based on the criteriae proposed by Edmonson and Steiner [36]. The tumors were staged according to the American Joint Committee on Cancer system [37]. Because the aim of our study was to evaluate the prognostic value of resectable HCCs, patients classified with stages IVA and IVB were excluded. The margins of the surgical specimens were inked and checked microscopically. Only completely resected specimens were included in our study.

### Differential Display

For differential display, paired mRNA samples from tumor cells and non-cancerous liver parenchyma cells from 3 low-stage (Stage I) and 6 high-stage HCCs were examined. Total RNA (2 µg) was reverse transcribed to produce cDNA using 500 units of Moloney murine leukemia virus reverse transcriptase (Bethesda Research Laboratories, Gaithersburg, MD, USA), the H-T11C primer (5'- AAGCTTTTTTTTTTTC-3'), 16 µM deoxynucleotide triphosphates (dNTPs), 16 µM DTT, 20 units of RNasin, and reverse transcription buffer in a 30-µL reaction volume for 60 min at 35°C. Polymerase chain reaction (PCR) was performed using 0.1 mL of reverse transcription reaction mixture and 1 unit DNA polymerase in a solution containing 2.5 µM H-T11C, 0.5 µM random primer HAP6 (5'-AAGCTTGCACCAT-3'), 1× PCR buffer, 8 mM dNTPs, and 10 µCi [-S^35^]dATP. The PCR procedure was performed using 40 cycles of 94°C for 30 s, 37°C for 1 min, and 72°C for 30 s, followed by a final elongation at 72°C for 5 min. After adding loading buffer to the reaction, the PCR products were heated at 70°C for 10 min, and separated by electrophoresis on a 6% polyacrylamide gel. Kodak XAR-5 film was exposed on the gels for 48 h. Bands that indicated the differential expression of mRNA were excised from the dried gel, eluted, re-amplified, cloned, and sequenced as previously described [3], [38], [39].

### RNA Isolation and Reverse Transcription–polymerase Chain Reaction (RT-PCR)

Total RNA was isolated from tissue samples and cell lines using the Trizol reagent (Life Technologies, Invitrogen, Carlsbad, CA. USA), according to the manufacturer’s instructions. RT-PCR was used to determine the mRNA levels of S100P in the paired HCC and non-tumorous liver samples. S26 ribosomal protein mRNA, a housekeeping gene, was used as an internal control. PCR was arrested during the exponential phase for each gene (i.e. 28 cycles for S100P and 22 for S26). PCR was performed in an automatic DNA thermal cycler (PerkinElmer, Wellesley, MA, USA) with initial heating at 94°C for 2 min followed by cycles at 94°C for 30 s, 58°C for 1 min, 72°C for 1 min and finally, 72°C for 10 min. The primers for S100P were S100P-F (5′-CTCAAGGATCTGATGGAGAA -3′) and S100P-R (5′- CCAGGGCATCATTTGAGTCC-3′). The primers for S26 were S26-F (5′- CCGTGCCTCCAAGATGACCAAAG-3′) and S26-R (5′-GTTCGGTCCTTGCGGGCTTCAC-3′).

### Immunohistochemical Analysis of S100P Protein Expression

The S100P protein was detected in formalin-fixed, paraffin-embedded sections of HCC and liver tissues using a labeled streptavidin-biotin method after antigen retrieval, as previously described [6], [11]. The tissue sections were dewaxed and rehydrated. The antigen was retrieved by incubating the slides in 0.01 M citrate buffer at 100°C for 10 min. After blocking with 3% H_2_O_2_ and 5% fetal bovine serum (FBS), the slides were incubated in a 1∶200 dilution of a goat anti-S100P polyclonal antibody (R&D Systems, Minneapolis, MN, USA) at 4°C overnight. The slides are then incubated with N-Histofine Simple Stain Mouse MAX PO (G) reagent (Cosmo Bio, Carlsbad, CA, USA). The peroxidase activity was visualized using a diamino-benzidine tetrahydroxychloride solution (BioGenex, San Ramon, CA, USA), and the sections were counterstained with hematoxylin. We replaced the primary antibody with 5% FBS as a negative control. One pathologist who was blinded to patients' outcomes calculated the percentage of positive cells based on 5 independent microscopic fields (400× magnification) for each slide. For data presentation, the proportion of the tumor cells that were positive for S100P immunostaining were categorized as diffuse S100P expression (>50%), focal or heterogeneous S100P expression (10% to 50%), or S100P expression in a small number of tumor cells (1% to 10%). In the nontumorous liver, S100P protein was detected only in very few isolated liver cells. Hence, HCC with more than 1% of tumor cells showing immunostaining for S100P was regarded as positive [32].

### Analysis of *p53* and *β-catenin* Mutations

Mutations of the *p53* tumor suppressor gene were analyzed in 187 tumors by direct sequencing of the chromosomal region spanning exon 2 to exon 11, as described previously [40]. Mutations of the *β-catenin* gene were analyzed in 214 cases by direct sequencing of exon 3 in the chromosomal DNA as described previously [34].

### Follow-up Examination and Early Tumor Recurrence

All patients had been followed up for more than 5 years or until death. Among the 305 study patients, 106 (34.6%) survived longer than 5 years, and 259 (84.9%) were eligible for the evaluation of ETR. The follow-up periods for survivors ranged from 30 to 236 months (median, 136 months). Following surgery, all patients received laboratory examinations, including assessment of serum α-fetoprotein (AFP) level, at 1- to 6-month intervals, and ultrasonography of the liver at 3- to 12-month intervals. Computed tomography (CT) and/or magnetic resonance imaging (MRI) were used to confirm and differentiate intrahepatic recurrence and/or distal metastasis in patients with clinical signs of recurrence. Cases of CT/MRI confirmed intrahepatic tumor recurrence or distant metastasis within 12 months of tumor resection were defined as ETR events, as previously described [6], [41].

Depending on the tumor site, the tumor size, the number of tumors, the level of liver function, and the patient's condition, tumor recurrence was treated by a second resection, percutaneous ethanol injection, transhepatic arterial chemo-embolization, radiofrequency ablation, or chemotherapy. All patients had an equal opportunity to access all therapeutic modalities supported by the Bureau of National Health Insurance, Taiwan.

### Statistical Analysis

The data analyses were performed using the Epi Info computer software, version 7.1.0.6 (Centers for Disease Control and Prevention, Atlanta, GA, USA). A univariate analysis was used to examine whether the immunohistochemical markers correlated with the gene mutations and the clinical and pathological parameters using the χ^2^ test. The survival rates after tumor resection were calculated using the Kaplan-Meier method, and the difference in the survival curves was analyzed using the log rank test. A multivariate survival analysis of all the parameters that were found to be significantly correlated in the univariate analysis was performed using a Cox proportional-hazards regression model. A two-tailed *P* value of less than 0.05 was considered to indicate a statistically significant relationship.

## Results

### Expression of S100P Protein in HCC and Liver Cells

**Figure 1:**
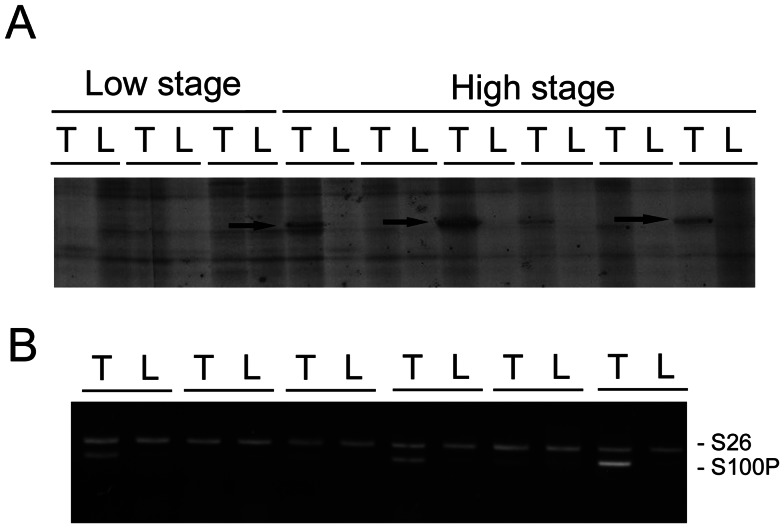
Identification and confirmation of overexpression of S100P in HCC. (A) Differential display showed that S100P expression was upregulated in high-stage HCCs. The arrows indicate bands of S100P, which was confirmed by subsequent cloning and sequencing. T: hepatocellular carcinoma; L: non-cancerous liver parenchyma. (B) Expression of S100P mRNA in paired HCC (T) and non-cancerous liver parenchyma (N). RT-PCR measurement identified S100P overexpression in 3 of 6 HCC specimens.

Using differential display, we identified a band overexpressed in high-stage HCC. The bands were excised from the gel and confirmed to be S100P by cloning and sequencing (Figure 1A). To prove S100P was overexpressed in HCC, the mRNA levels were measured in paired HCC and non-cancerous liver parenchyma. As shown in Figure 1B, S100P mRNA was overexpressed in 3 of 6 HCCs.

**Figure 2:**
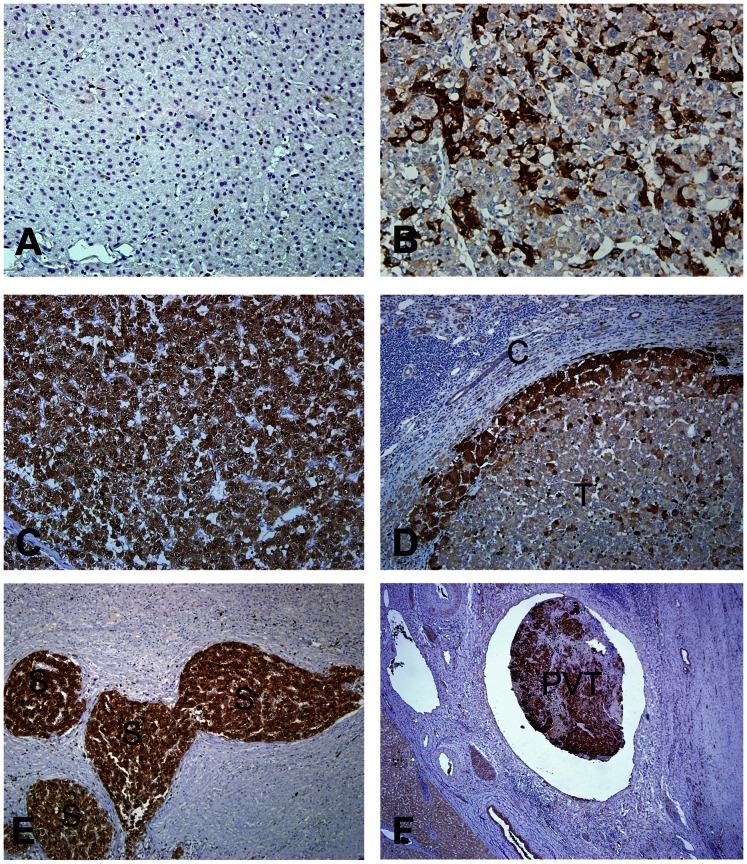
Expression of S100P in HCC and non-cancerous liver parenchyma. (A) No immunostaining of S100P in non-cancerous liver parenchyma. (B and C) Heterogeneous and diffuse expression of S100P in HCC. (D) The expression of S100P was more prominent at the periphery of the tumor mass near the tumor capsule. (E) Strong expression of S100P in satellite nodules. (F) A portal vein tumor embolus exhibiting strong S100P expression. T: tumor, C: capsule, S: satellite nodule, PVT: portal vein tumor embolus.

The immunohistochemical staining was used to screen 305 HCCs to determine the frequency of S100P expression and the clinical and pathological significance of S100P expression in HCC. S100P protein was not detected or detected only in very few isolated liver cells (Figure 2A), but was detected in the tumor cell cytoplasm and/or nuclei in 173 of 305 HCCs (56.7%). The level of S100P protein expression in HCC cells varied considerably, and displayed a heterogeneous distribution. S100P was expressed in a small number of tumor cells in 131 cases. Heterogeneous to focal S100P expressions in 10∼50% tumor cells were seen in 33 cases (Figure 2B). Diffuse S100P expressions in more than 50% tumor cells were seen in 9 cases (Figure 2C). In cells that exhibited heterogeneous to focal or trace S100P expression, S100P was predominantly expressed at the periphery of the tumor masses (Figure 2D) and satellite nodules (Figure 2E). Notably, the intravascular tumor thrombi in large and small portal vein branches often showed more intense and diffuse immunoreactivity than the main tumor mass (Figure 2F). In about 10% of all cases, the S100P protein was detected in a small subset of hepatocytes that usually comprised less than 1% of the total hepatocytes. Therefore, those that did not express S100P or expressed S100P in less than 1% of the tumor cells were defined as the negative group (132 cases). In some specimens, S100P was also expressed in inflammatory cells that were located within both tumors and non-cancerous liver tissues.

### Clinical and Pathological Significance of S100P Expression in HCC

**Figure 3:**
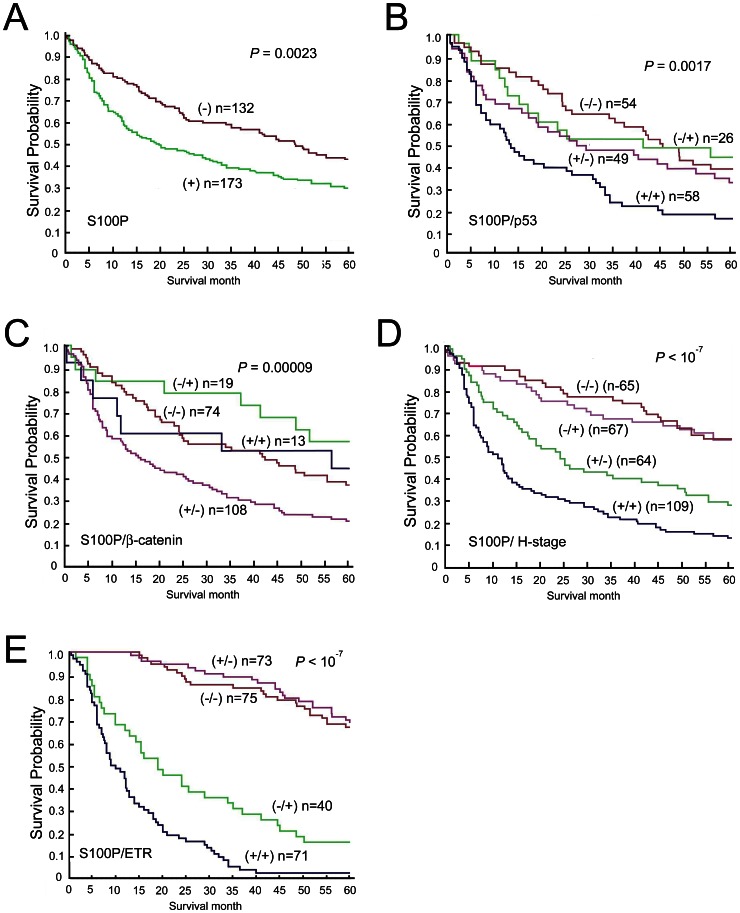
Kaplan–Meier analysis of overall survival in 305 patients with HCC. (A) The expression of S100P protein in HCC tumor cells was associated with a significantly lower 5-year survival rate than that of HCC tumors in which the S100P protein was not expressed. (B) Patients with S100P-positive, *p53*-mutated HCCs had a lower 5-year survival rate than the other groups. (C) Patients with S100P-positive, *β-catenin*-wild type HCCs had the lower 5-year survival rate than the other groups. (D) High tumor stage and concurrent S100P expression in HCC patients were associated with the lower 5-year survival than that of HCC patients with high stage but S100P negative tumors. (E) ETR and concurrent S100P expression in HCC patients were associated with lower 5-year survival than that of HCC patients with ETR but S100P negative tumors. (+): The presence of S100P expression, p53 mutation, β-catenin mutation, high tumor stage, or ETR. (−): The absence of S100P expression, p53 mutation, β-catenin mutation, high tumor stage, or ETR.

To elucidate the significance of S100P expression in HCC, we examined possible correlations between S100P protein expression and major clinical and pathological features of HCC. As shown in Table 1, S100P protein expression tended to occur in female patients (*P = *0.0162), and correlated with high serum AFP (>200 ng/mL; *P = *0.0001). However, it did not correlate with other clinical parameters, such as age, serum HBsAg, Anti-HCV status, or tumor size. S100P expression closely correlated with high tumor grade (grade 2 to 4; OR, 2.12; 95% CI, 1.25–3.62; *P = *0.0029) and high tumor stage (stages II and III; OR, 1.65; 95% CI, 1.02–2.69; *P = *0.0319). The Kaplan–Meier survival analysis showed S100P expression was associated with lower 5-year survival in HCC patients (Figure 3A).

**Table 1:** Univariate analysis of S100P protein expression with various clinicopathological features and aberrant gene expression in 305 patients with surgically removed unifocal primary hepatocellular carcinoma.

	S100P protein expression			
Variables	Total	Yes n (%)	Odds Ratio	*P* value
Age				
>56	136	75 (55)	1.0	
≤56	169	98 (58)	1.12 (0.69–1.82)	0.6186
Sex				
Male	239	127 (53)	1.0	
Female	66	46 (70)	2.03 (1.09–3.79)	0.0162
HBsAg				
Negative	103	61 (59)	1.0	
Positive	202	112 (55)	1.17 (0.80–1.53)	0.5889
Anti-HCV				
Negative	184	97 (53)	1.0	
Positive	97	59 (61)	1.39 (0.82–1.32)	0.1935
α-fetoprotein (ng/ml)				
≤200	167	78 (44)	1.0	
>200	138	95 (69)	2.52 (1.53–4.16)	0.0001
Tumor size (cm)				
≤5	149	77 (52)	1.0	
>5	156	96 (62)	1.50 (0.92–1.54)	0.0823
Tumor grade				
1	203	103 (51)	1.0	
2∼4	102	70 (69)	2.12 (1.25–3.62)	0.0029
Tumor stage				
I	129	64 (50)	1.0	
II∼III	176	109 (62)	1.65 (1.02–2.69)	0.0319
*p53* mutation				
No	103	49 (48)	1.0	
Yes	84	58 (69)	2.46 (1.29–4.71)	0.0032
*β-catenin* mutation				
No	182	108 (59)	1.0	
Yes	32	13 (41)	0.46 (0.20–1.07)	0.0489
Early tumor recurrence				
No	115	40 (35)	1.0	
Yes	144	71 (49)	1.82 (1.10–3.02)	0.0189

### S100P Expression Predicts ETR and Poor Prognosis

The ETR is the most critical clinical factor that is predictive of poor prognosis in HCC after hepatectomy [6], [41]. In our series, ETR occurred in 111 of 259 patients (42.9%). ETR occurred at a rate that was approximately 2 times higher in patients with S100P-positive HCCs than those with S100P-negative HCCs. (*P = *0.0198; Table 1). To further elucidate the factors associtaed with ETR, we investigated its relationship with the major clinical and pathological factors. We found that serum HBsAg (*P = *0.0045), tumor size (*P = *1.2×10^−6^), tumor grade (*P = *0.0456), and tumor stage (*P*<1×10^−7^) were important clinical and histopathological risk factors for ETR (Table 2). Four molecular markers were also analyzed. A high serum level of AFP (*P = *2.3×10^−6^), the presence of the *p53* mutation (*P = *0.0032), and the absence of the *β-catenin* mutation (*P = *0.0011) predicted ETR. Notably, S100P expression was also a significant risk factor for ETR (*P = *0.0189) (Table 2).

**Table 2:** Univariate analysis of clinicopathological variables and S100P protein expression with early tumor recurrence (ETR) in patients with surgical removed unifocal primary hepatocellular carcinoma.

	ETR			
Variables	Total	Yes n (%)	Odds Ratio	*P* value
**Clinical features**				
Age				
>56	114	41 (43)	1.0	
≤56	145	70 (48)	1.24 (0.73–2.09)	0.3961
Sex				
Male	201	85 (42)	1.0	
Female	58	26 (45)	1.11 (0.59–2.08)	0.7307
HBsAg				
Negative	88	27 (31)	1.0	
Positive	171	84 (49)	2.18 (1.22–3.90)	0.0045
Anti-HCV				
Negative	163	74 (45)	1.0	
Positive	80	30 (38)	1.39 (0.77–2.49)	0.2422
**Histopathological features**				
Tumor size (cm)				
≤5	130	34 (26)	1.0	
>5	129	77 (60)	4.18 (2.47–7.07)	1.2×10^−6^
Tumor grade				
1	176	68 (38)	1.0	
2∼4	83	43 (52)	1.70 (1.01–2.89)	0.0456
Tumor stage				
I	114	19 (17)	1.0	
II∼III	145	92 (63)	8.68 (4.78–15.77)	<1×10^−7^
**Molecular markers**				
α-fetoprotein (ng/ml)				
≤200	144	43 (30)	1.0	
>200	115	68 (59)	3.40 (1.97–5.89)	2.3×10^−6^
*p53* mutation				
No	89	30 (34)	1.0	
Yes	74	42 (57)	2.58 (1.30–5.14)	0.0032
*β-Catenin* mutation				
No	158	79 (50)	1.0	
Yes	26	4 (15)	5.50 (1.81–16.69)	0.0011
S100P expression				
No	115	40 (35)	1.0	
Yes	144	71 (49)	1.82 (1.07–3.12)	0.0189

To elucidate whether S100P is an independent factor for predicting patient survival, high tumor stage, and ETR, multivariate analyses using a Cox proportional-hazards model were performed (Table 3). We found that ETR (*P*<0.0001), high tumor stage (*P = *0.0256), and S100P expression (*P* = 0.0044) were independent risk factors for poor survival in HCC patients.

**Table 3:** Multivariate analyses of prognostic factors in hepatocellular carcinoma patients.

Covariate	Coefficient	S.E.	Z-Statistic	H.R. (95% C.I.)	*P-* value
Sex (M/F)	0.0143	0.2479	0.0577	1.0144 (0.6241–1.6489)	0.9540
AFP (L/H)	0.0628	0.2262	0.2777	1.0648 (0.6834–1.6591)	0.7813
Grade (L/H)	−0.2358	0.2227	−1.0586	0.7895 (0.5106–1.2223)	0.2898
Stage (L/H)	−0.6438	0.2883	−2.2329	0.5253 (0.2985–0.9243)	0.0256
*p53* mutation (P/N)	−0.0743	0.2263	−0.3282	0.9284 (0.5958–1.4467)	0.7427
*β-catenin* mutation (P/N)	−0.0149	0.3779	−0.0393	0.9853 (0.4698–2.0663)	0.9686
ETR(P/N)	2.1589	0.2690	8.0264	8.6620 (5.1128–14.6748)	<0.0001
S100P expression (P/N)	0.6481	0.2277	2.8459	1.9118 (1.2235–2.9873)	0.0044

Abbreviations*:* S.E., Standard error; H.R., Hazard ratio; C.I., Confidence interval; M, male; F, female; AFP,

α-fetoprotein; ETR, early tumor recurrent; P, presence; N, absence; L: low; H: high.

### Correlation of S100P Expression with *p53* and *β-catenin* Mutations

The *p53* and *β-catenin* genes are commonly mutated in HCCs [34], [40]. In our HCC series, the *p53* mutation was detected in 84 of 187 cases (44.9%), and the *β-catenin* mutation was identified in 32 of 214 tumors (15.0%). As shown in Table 1, S100P protein expression correlated with the *p53* mutation (*P = *0.0032), and the absence of the *β-catenin* mutation (*P = *0.0489). To better understand the role of S100P expression in the progression of HCC, we stratified HCC patients according to S100P, *p53* and *β*-catenin gene status. As shown in Table 4, HCCs with S100P expression and *p53* mutation had the highest frequencies of high-stage tumor (stage II and III) and ETR (88% and 69% of cases, respectively), which were approximately 2-fold higher than the rates of high-stage tumor and ETR in HCC cases without either S100P expression or the *p53* mutation (44% and 35%; *P = *1×10^−6^ and *P = *0.00068; respectively). Hence, HCC patients with S100P expression and *p53* mutation had the lowest 5-year survival (*P = *0.0017 (Figure 3B), even worse than those with the *p53* mutation (+)/S100P (-) (*P* = 0.01) or *p53* mutation (-)/S100P (+) tumors (*P = *0.01 and *P = *0.035, respectively).

**Table 4:** Interaction between S100P expression with *p53* mutation or *β-catenin* mutation in the tumor progression of hepatocellular carcinoma.

Feature	S100P expression/p53 mutation				
	Yes/Yes	Yes/No	No/Yes	No/No	*P* value
Stage					
I	7 (12%)^a^	23 (47%)	9 (35%)	30 (56%)^a^	0.0000115
II–III	51 (88%)^b,^	26 (53%)^b^	17 (65%)	24 (44%)	
ETR†					
Presence	35 (69%)^c^	13 (33%)^c^	7 (32%)	17 (35%)	0.00051
Absence	16 (31%)^d^	27 (67%)	15 (68%)	32 (65%)^d^	
	S100P expression/*β-catenin* mutation				
	**Yes/Yes**	**Yes/No**	**No/Yes**	**No/No**	*P* value
Stage					
I	7 (54%)^e^	27 (25%)^e^	15 (79%)	28 (38%)	0.0000446
II–III	6 (46%)	81 (75%)^f^	4 (21%)^f^	46 (62%)	
ETR†					
Presence	2 (22%)	53 (57%)^g^	2 (12%)^g^	26 (40%)	0.00142
Absence	7 (78%)^h^	40 (43%)^h^	15 (88%)	39 (60%)	

Abbreviations: NS, not significant; ETR, early tumor recurrence.

† Tumor recurrence within 12 months after hepatectomy.

a, b, c, d, e, f, g and h designate comparison between the indicated two groups.

*P* values: ^a^0.000001; ^b^0.00006; ^c^0.0006; ^d^0.00068; ^e^0.0288; ^f^0.000004; ^g^0.0015; ^h^0.0457.

In contrast to the *p53* mutation, the *β-catenin* mutation is associated with low tumor grade, low tumor stage, and better 5-year survival in HCC [34]. Consistent with such findings, our data showed a correlation between S100P expression and the absence of the *β-catenin* mutation in HCC tumors (Table 1, *P = *0.0489). In addition, as shown in Table 4, *β-catenin*-wild type HCCs with S100P expression had the highest frequencies of high tumor stage (75%) and ETR (57%), followed by HCC without S100P expression or the *β-catenin* mutation (62% and 40%, respectively). HCC with the *β-catenin* mutation but negative for S100P expression had lowest risk of high-stage tumor and ETR (21%, *P = *0.0000446 and 12%, *P = *0.00142, respectively). Hence, HCC patients with S100P expression and absence of *β*-catenin mutation had the lowest 5-year survival (Figure 3C), than those with *β*-catenin-mutated HCC or S100P (+), *β*-catenin-wild type HCC.

### S100P Expression Predicts Poor Prognosis in Patients with High-stage Tumors or ETR

Because tumor stage and ETR are most important predictive factors for poor prognosis in HCC, we further analyzed the prognostic role of S100P expression in patients with high tumor stage or ETR. The combinatorial analysis showed that HCC patients with high tumor stage and S100P expression had a significantly lower -5-year survival rate (Figure 3D) than high-tumor-stage HCC patients without S100P expression (*P = *0.0026). Similarly, HCC patients with ETR and S100P expression had a significantly lower 5-year survival rate (Fig. 3E) than HCC patients with ETR alone (*P = *0.0002).

## Discussion

Although S100P was reported to be expressed in various types of human cancer cells [13], [15], [19], [24]–[32], including human HCC [33], the clinical and pathological significances of S100P expression in HCC remains largely unclear. We found that S100P expression correlated with high tumor grade, high serum AFP level (>200 ng/mL), and large tumor size (>5cm). Dowen et al. reported that S100P expression correlated significantly with increasing grade of pancreatic intraepithelial neoplasia [42]. In addition, the down-regulation of S100P expression by small interfering RNA treatment suppressed growth and increased cellular apoptosis in Hep3B cells [33]. The knockdown of S100P expression decreased the S-phase fraction of cisplatin sensitive cell lines and slow cell proliferation [43]. These findings suggest that S100P plays an important role in facilitating tumor cell proliferation and differentiation, and S100P expression in HCC facilitates tumor cell growth and contributes to large tumor size, poor differentiation, and high AFP level.

Intrahepatic tumor spread through the portal vein system is the most crucial histological feature for high-stage HCC and is associated with poor prognosis [3]. However, the molecular markers related to vascular invasion of HCC are poorly understood. We demonstrated that high tumor stage (stage II and III) in HCC, which had vascular invasion and various degrees of intrahepatic spread, was more frequently associated with S100P expression, compared with HCCs of low tumor stage (stage I). In addition, patients with S100P-negative HCCs had better 5-year survival than patients with S00P-positive HCCs.

We also showed that S100P protein immunoreactivity was more intense at the tumor border, and that it was more diffuse and intense in satellite nodules and portal vein tumor emboli than in the main tumor mass. Arumugam et al. reported that S100P expression correlated with cell proliferation, migration, and invasion in pancreatic cancer [44]. Du et al. reported that the induction of S100P expression resulted in significantly reduced cellular adhesion and enhanced cell migration [45]. Chandramouli et al. demonstrated that the knockdown of S100P expression compromised invadopodia formation, colony growth, and cellular motility in colon cancer [46], and Zhou et al. showed that S100P plays a critical role in conferring tamoxifen resistance and enhancing cell motility [47]. These findings suggest that S100P expression is an important factor for tumor invasiveness and the metastatic potential of HCC, and may thus be associated with high tumor stage and poor prognosis.

To further elucidate the impact of the expression of S100P on the incidence of ETR, we investigated possible relationships with the major clinical and pathological factors. We found that large tumor size, high tumor grade, and high tumor stage were significant histopathological predictors of ETR, and that high AFP, the presence of the *p53* mutation, and the absence of the *β-catenin* mutation were significant molecular factors for ETR. In addition, we demonstrated for the first time that HCCs with S100P expression were associated with a higher risk for ETR than HCCs that did not express S100P. The expression of S100P has also been shown to be a potential prognostic biomarker in colorectal cancer [48], breast cancer [49], and diffuse large B cell lymphoma [50]. These findings suggest that HCCs with S100P expression may have enhanced invasion/metastasis potential, thus contributing to more frequent ETR and poor prognosis.

In addition, the association of S100P with other molecular features, especially the *p53* and *β-catenin* mutations [4], [34], [40], requires clarification. Mutations of the *p53* and *β-catenin* genes contribute to two distinct pathways of hepatocarcinogenesis [51]. Inactivation of *p53* leads to aberrant mitosis, chromosome instability, more aggressive tumorigenesis, and poor prognosis in HCC [4], [35], whereas the *β-catenin* mutation is associated with low tumor grade and low tumor stage, and better patient survival [34]. We showed that S100P expression correlated with the presence of the *p53* mutation and the absence of the *β-catenin* mutation. Therefore, we conducted a combinatorial analysis to further elucidate the clinical significance of the mutations with regard to S100P expression. Our findings suggest that S100P expression is associated with HCC progression, and it interacts positively with the *p53* mutation, contributing to more advanced disease. This observation is similar to our previous report that stathmin expression interacts positively with the *p53* mutation, and contributes to advanced HCC [6]. Thus, S100P expression is an important molecular prognosticator of HCC, and contributes to poor prognosis when concurrent with the *p53* mutation.

We also showed that HCC with S100P expression alone had 3- and 5-fold higher frequencies of vascular invasion (75%) and ETR (57%), than those observed for HCC with the *β-catenin* mutation and absence of S100P expression, which had the lowest frequency of vascular invasion (21%) and ETR (12%). Although HCC with S100P expression and the *β-catenin* mutation was rare in our series, occurring in only 6% of our cases, we found that the incidences of vascular invasion and ETR were higher in HCCs with S100P expression and wild type *β-catenin*. Further analysis showed that the majority of HCC cases with the *β-catenin* mutation had low tumor stage and a lower incidence of ETR, regardless of the presence or absence of S100P expression (7 of 13 versus 15 of 19 and 2 of 9 versus 2 of 17, respectively, both *P*>0.05). These findings suggest that mutant *β-catenin* exerts a strong negative effect on tumor progression that is not abolished by S100P expression. Taken collectively, these findings indicate that S100P expression and the *β-catenin* mutation play opposing roles in vascular invasion and ETR in HCC. These data highlight the importance of combinatorial analysis to better understand the significance of interactions between different molecular factors in disease processes, particularly in tumor progression.

Our previous studies have shown that tumor stage and ETR are independent prognostic factors in HCC [7], [11]. These findings encouraged us to investigate possible prognostic factors in HCC patients with high tumor stage and ETR. We found that S100P expression is an independent prognostic factor in HCC patients with high tumor stage and ETR. We found that HCC patients with high tumor stage and S100P expression had a lower 5-year survival rate than high tumor stage HCCs without S100P expression. Similarly, HCC patients with ETR and S100P expression had a lower 5-year survival rate than patients with S100P-negative HCC and ETR. These findings indicated that the expression of S100P exerted additional adverse effects on survival in HCC patients with high tumor stage or ETR. Thus, the stratification of patients into groups based on clinical and pathological factors may lead to a more accurate prediction of patient survival, and may aid in the development of better management strategies. Moreover, our findings suggest that the expression of S100P augments the metastatic potential of HCCs, resulting in high tumor stage, and increases the severity of the disease course, contributing to poor prognosis in HCC patients with high tumor stage or ETR.

In conclusion, our study revealed in vivo evidence that the expression of S100P is an important molecular factor for vascular invasion and intrahepatic spread, and may represent a predictive biomarker for ETR, and thus a prognosticator of unfavorable outcome. In addition, our combinatorial analysis revealed an additive unfavorable prognostic interaction of S100P expression and the *p53* mutation. These findings also highlight the potential importance of combinatorial analyses for the molecular features of HCC. The expression of S100P may identify HCC patients with high tumor stage or ETR that are at increased risk for reduced survival, aiding in the development of improved management strategies.
